# Anatomy and Cytogenetic Identification of a Wheat-*Psathyrostachys huashanica* Keng Line with Early Maturation

**DOI:** 10.1371/journal.pone.0131841

**Published:** 2015-10-13

**Authors:** Liangming Wang, Yang Liu, Wanli Du, Fan Jing, Zhonghua Wang, Jun Wu, Xinhong Chen

**Affiliations:** Shaanxi Key Laboratory of Plant Genetic Engineering Breeding, College of Agronomy, Northwest Agriculture and Forestry University, Yangling, Shaanxi, China; Sabanci University, TURKEY

## Abstract

In previous studies, our research team successfully transferred the Ns genome from *Psathyrostachys huashanica Keng* into *Triticum aestivum* (common wheat cv. 7182) using embryo culture. In the present study, one of these lines, i.e., hybrid progeny 25-10-3, which matured about 10–14 days earlier than its wheat parent, was assessed using sequenced characterized amplified region (SCAR) analysis, EST-SSR and EST-STS molecular markers, and genomic in situ hybridization (GISH). We found that this was a stable wheat-*P*. *huashanica* disomic addition line (2*n* = 44 = 22 II) and the results demonstrated that it was a 6Ns disomic chromosome addition line, but it exhibited many different features compared with previously characterized lines, i.e., a longer awn, early maturation, and no twin spikelets. It was considered to be an early-maturing variety based on the early stage of inflorescence initiation in field experiments and binocular microscope observations over three consecutive years. This characteristic was distinct, especially from the single ridge stage and double ridge stage until the glume stage. In addition, it had a higher photosynthesis rate and economic values than common wheat cv. 7182, i.e., more spikelets per spike, more florets per spikelet, more kernels per spike, and a higher thousand-grain weight. These results suggest that this material may comprise a genetic pool of beneficial genes or chromosome segments, which are suitable for introgression to improve the quality of common wheat.

## Introduction

Time management is a major issue for grain crops, where the transition from vegetative growth to reproductive growth is a critical period for successful reproduction, and thus it is important that it occurs when the internal and external conditions are appropriate [1]. Due to the joint pressures of environmental change and artificial selection, common wheat (*Triticum aestivum* L., 2*n* = 6*x* = 42, AABBDD) evolved into two varieties: winter wheat and spring wheat [2], where the former requires a period of low temperature or vernalization whereas the latter does not [1,3,4]. Thus, winter wheat needs to be planted in areas where the winter is cold because it requires low temperature conditions [5]. However, due to its narrow genetic background and genetic diversity, the yield of bread wheat is often reduced significantly by biotic or abiotic stresses [6]. The heading stage is a very important period in the wheat growth process and it is employed as a major index to determine whether the wheat period growth duration is short or long. Negative factors such as drought, rain, high temperature, and preharvest sprouting [7] are severe problems in agriculture, which comprise the leading causes of yield reduction and quality declines in wheat production in some areas, especially in Huang-Huai-Hai and the low valley of the Yangtze River [8], one of the winter wheat-planting regions of China where a double cropping system is implemented. These areas experience rainy weather during June, which is the harvest time for wheat [9], so it is very important to develop an early-maturing variety to address this problem. The development of a suitable variety could avoid these difficult environmental conditions and facilitate the sowing of the next crop.

*Psathyrostachys huashanica* Keng (2*n* = 2*x* = 14, NsNs) is a wild relative of common wheat, which is only found on Huashan Mountain, a branch of the Qinling Mountain Range in Shaanxi province, China. It possesses many potentially valuable traits such as abiotic stress tolerance, resistance to disease, and early maturation [10]. Wheat-*P*. *huashanica* 6Ns disomic addition line was developed and compared in a previous study [11]. 25-10-3, a new Wheat-*P*. *huashanica* 6Ns disomic addition line was found, and this study showed that they shared some of the same molecular markers while their morphological and physiological characteristics were different. The aims of the present study were as follows: a) to identify 25-10-3 using *P*. *huashanica*-specific sequenced characterized amplified region (SCAR) analysis, and EST-SSR and EST-STS markers; b) to verify the chromosomal configuration of progeny line 25-10-3 using genomic in situ hybridization (GISH); c) to characterize photosynthesis in 25-10-3; and d) to evaluate the early maturation of 25-10-3 based on its anatomy.

## Materials and Methods

### Plant materials

Disomic addition line 25-10-3 (2*n* = 44) and its parents, i.e., common wheat cv. 7182 (2*n* = 6*x* = 42, AABBDD) and *P*. *huashanica* (2*n* = 2*x* = 14, NsNs), were used in this study. All of these materials were created by Shaanxi Key Laboratory of Genetic Engineering for Plant Breeding, College of Agronomy, Northwest A&F University, Shaanxi, China [12]. Addition line 25-10-3 and common wheat cv. 7182 were sown in 50 rows at a row distance of 20 cm with 10 seeds per row. Sowing and sampling were conducted on the same days each year, i.e., October 2010 to June 2011; October 2011 to June 2012; and October 2012 to June 2013. All of materials were sown at experimental field of College of Agronomy, Northwest A&F University, Yang ling (at 108° E, 34° N), Shaanxi, China, and conventional management was applied.

### GISH analysis

GISH was used to determine the chromosomal composition and configuration of 25-10-3 in root tips and pollen mother cells (PMCs). The total genomic DNA of *P*. *huashanica* was extracted from fresh leaves using the modified cetyltrimethylammonium bromide method [13]. The DNA probe for *P*. *huashanica* was labeled with digoxigenin-11-dUTP (Roche, Germany) via the nick-translation method. GISH was performed as described previously [14].

### SCAR molecular analysis of Ns in *P*. *huashanica*

Genomic DNA was extracted from 25-10-3 and its parents, as described previously [13]. Two pairs of specific markers (RHS23 and RHS141) were used to detect the disomic addition line 25-10-3 and its parents, i.e., common wheat cv. 7182 and *P*. *huashanica*. Full details of the specific markers for *P*. *huashanica* have been described previously [15,16]. The PCR amplification mixture comprised 2 μL primer (2.5 mM), 2 μL DNA template (50–100 ng/μL),1.6 μL dNTPs (2.5 mM), 0.2 μL Taq polymerase (5 U/μL), 1.6 μL MgCl_2_ (2.5 M), 2 μL 10× PCR buffer, and doubled distilled (DD) H2O up to a final volume of 20 μL. The amplified products were resolved by polyacrylamide gel electrophoresis (PAGE) using 1% agarose gels, followed by ethidium bromide staining under UV light and photographic images were captured.

### PAGE of EST-SSR and EST-STS markers

Genomic DNA from the three lines was purified as described previously [13]. We screened 1200 pairs of multiple loci primers, including EST-SSRs and EST-STSs, which belong to the seven homoeologous groups of wheat, including 120 pairs which belong to the sixth homoeologous group, thereby identifying the genomic composition of the wheat-*P*. *huashanica* addition line 25-10-3. Each reaction mixture had a total volume of 20 μL, which comprised 2 μL DNA template (50 ng/μL), 2 μL 10× PCR buffer, 1.6 μL dNTPs (2.5 mM), 2 μL primer (2.5 mM), 1.6 μL MgCl_2_ (2.5 mM), 0.2 μL Taq polymerase (5 U/μL), and 10.6 μL ddH_2_O. The PCR amplification procedure comprised initial denaturation at 94°C for 3 min, followed by 35 cycles for 60 s at 94°C, 50 s at 60°C, and 60 s at 72°C, with a final extension for 10 min at 72°C. The PCR amplification reaction products were analyzed by PAGE (8% gel) at a constant voltage (150 V) and visualized using silver nitrate.

### Sample and spike primordium development identification

Samples were obtained from five plants on each sampling every 7 days between the start of sowing until overwintering commenced, and every two weeks subsequently until the wheat became green in the following year from 2010 to 2013 respectively. The spike primordium anatomy was examined using a binocular dissecting microscope and photographs were captured with a digital camera (SMZ1500 NIKON).

### Photosynthetic indices

The photosynthesis rate, stomatal conductance, transpiration rate, and intercellular CO_2_ concentration, were determined at the grain filling stage using an LI-6400 Portable Photosynthesis System (USA) from 2010 to 2013 respectively., light source regulated1400μmol·m^-2^·s^-1^, open flow gas exchange, CO_2_ concentration regulated 365μmol·L^-1^. Each measurement 10 plants, average the results

### Analysis of agronomic traits

Plant height, tillering, spike length, kernel number per spike, spikelet number per spike, and thousand-kernel weight from 2010 to 2013 respectively, each measurement 10 plants, average the results. The Student’s *t*-test was used to determine significant differences between common wheat cv.7182 and 25-10-3.

## Results

### Cytogenetic and molecular characterization identification of the wheat-*P*. *huashanica* progeny line 25-10-3

**Fig 1:**
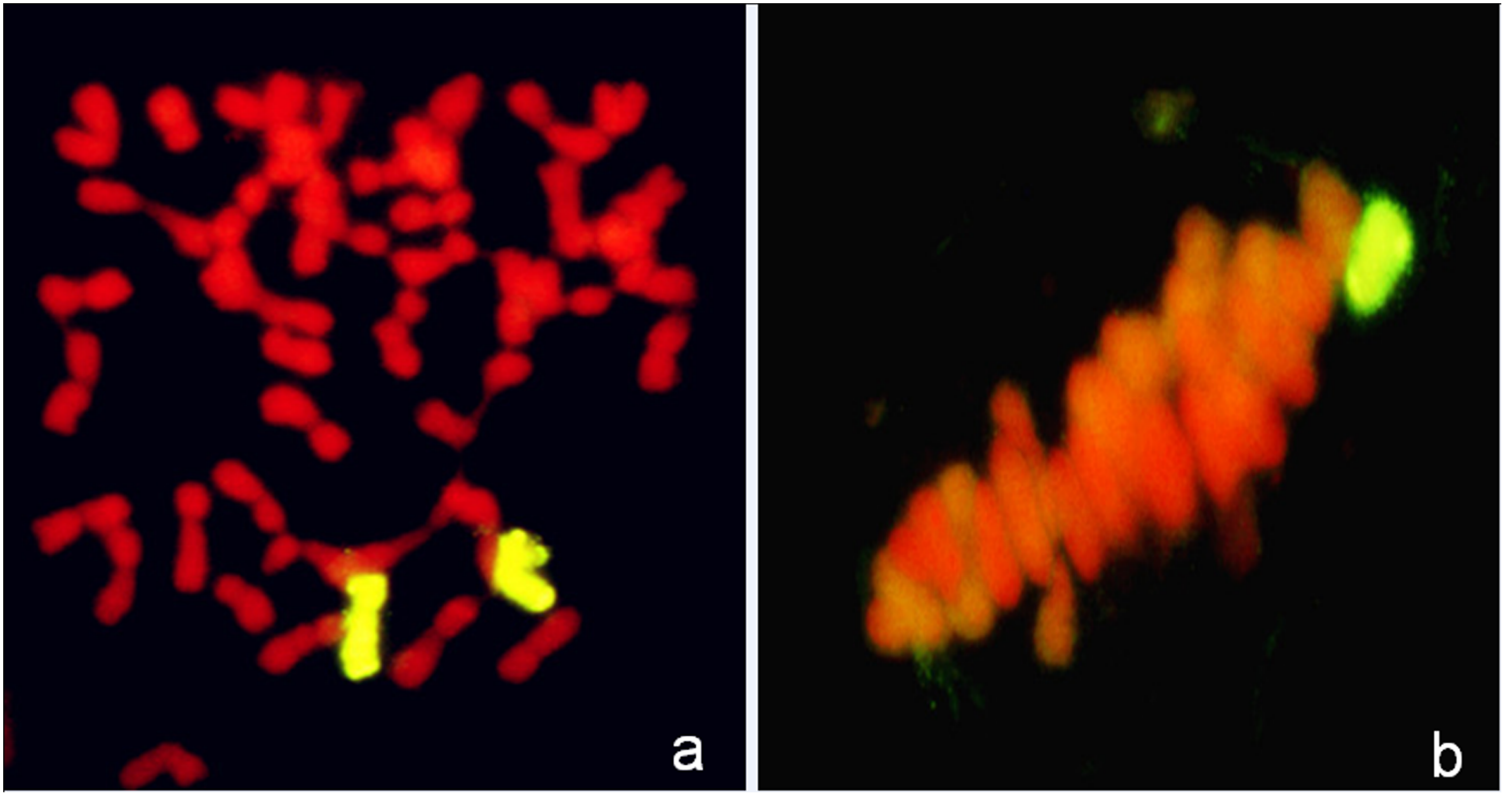
Genomic *in situ* hybridization (GISH) identification of the mitotic and meiotic patterns in wheat-*P*. *huashanica* disomic addition line 25-10-3. (a) Somatic chromosomes in the root tips: 2*n* = 44, i.e., 42 wheat chromosomes and two *P*. *huashanica* chromosomes with strong yellowish-green hybridization signals. (b) Chromosome behavior of pollen mother cells during metaphase I, i.e., 2*n* = 22 II, where the alien chromosome comprises one ring bivalent with a yellowish-green signal.

**Fig 2:**
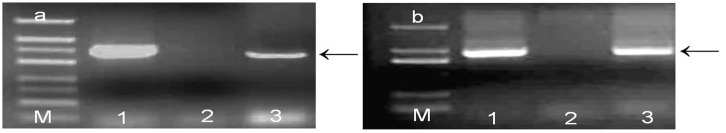
Analysis of the Ns genome of *P*. *huashanica* using SCAR markers. (a) RHS23, (b) RHS141; M, marker; 1, *P*. *huashanica*; 2, common wheat cv. 7182; 3, 25-10-3. Amplified specific bands in the *P*. *huashanica* using and addition line25-10-3 respectively (arrows).

**Fig 3:**
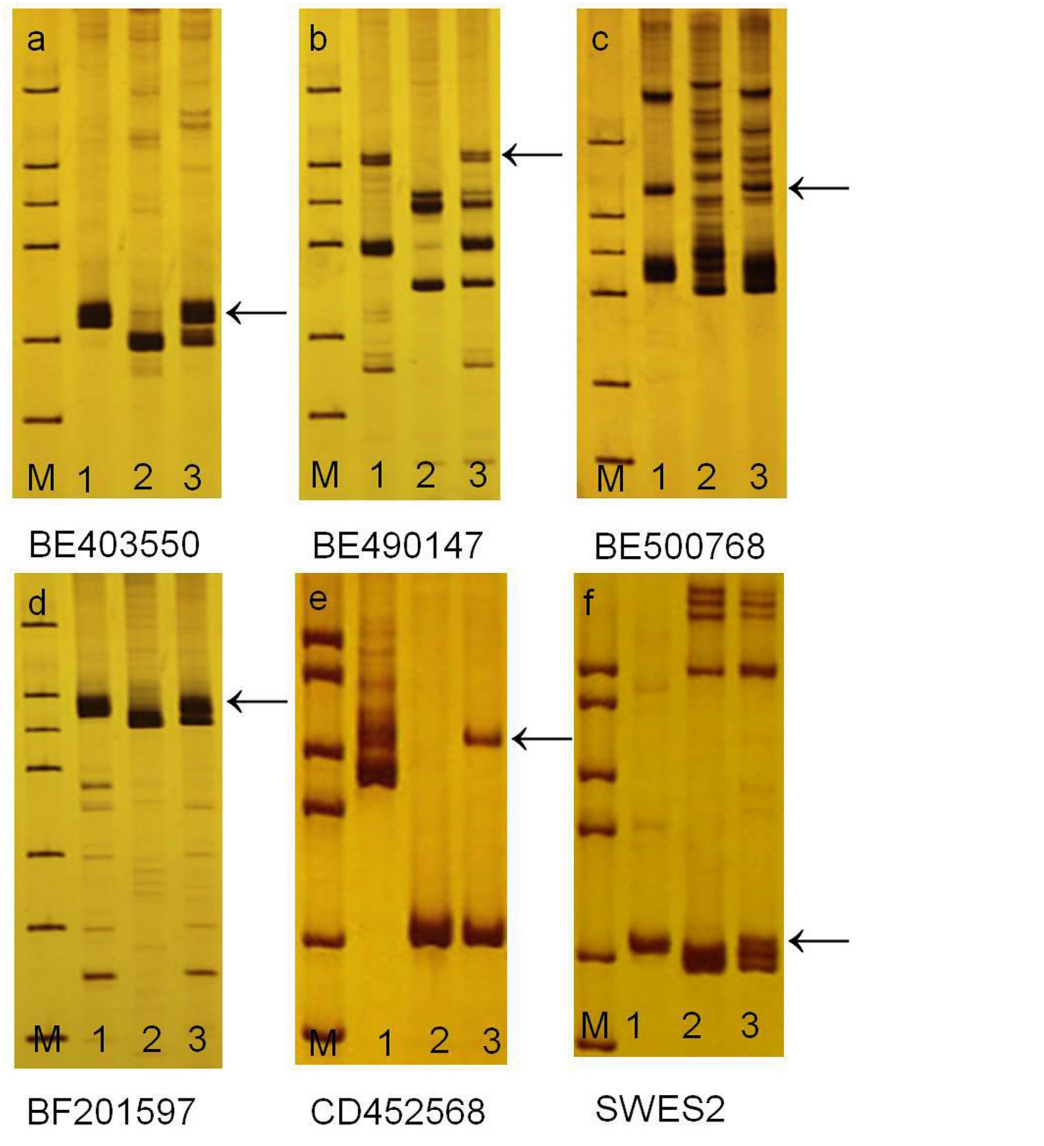
Analysis of addition line 25-10-3 and its parents, i.e., common wheat cv. 7182 and *P*. *huashanica*, using EST-SSR and EST-STS marker. Amplified specific bands in the addition line in chromosomes 6A, 6B, and 6D, respectively (arrows). EST-SSR marker: (a) BE403550; (b)BE490147; (c)BE500768; (d)BF201597; (e)CD452568; EST-STS marker: (f)SWES2; M, marker; 1, *P*. *huashanica*; 2, common wheat cv. 7182; 3, 25-10-3.

GISH analysis on mitotic root tip cells and metaphase I PMCs indicated that there were two chromosomes with strong yellowish-green hybridization signals and one yellowish-green bivalent ring (Fig 1a and 1b). The results confirmed that 25-10-3 contained 44 chromosomes, i.e., 42 wheat chromosomes and two *P*. *huashanica* chromosomes in the mitotic root tip cells. There were 22 II bivalents in the PMCs, i.e., 21 wheat bivalents and one *P*. *huashanica* bivalent. These results demonstrate that 25-10-3 is a stable *P*. *huashanica* disomic addition line, which has a pair of sister chromosomes that can pair and segregate normally during synapsis. The Ns genome-specific SCAR markers RHS 23 and RHS141 from *P*. *huashanica* were used to test the three materials. In *P*. *huashanica* and 25-10-3, 900 bps and 936 bps specific bands were amplified, respectively, but these bands were absent from the other parent, common wheat cv. 7182, as shown in Fig 2 and Table 1. These results confirmed that 25-10-3 possessed the Ns chromosomes of *P*. *huashanica*. 1200 pairs of EST-SSR and EST-STS primers related to all the homoeologous groups of wheat to determine the homoeologous relationships between wheat, *P*. *huashanica*, and 25-10-3. These comprised one EST-SSR SWES2 primer and five EST-STS pairs, i.e., BE403550, BE490147, BE500768, BF201597and CD452568, which were originally located on 6A, 6B, and 6D of common wheat. Our results showed that *P*. *huashanica* and 25-10-3 could amplify different allele-specific bands compared with common wheat cv. 7182 (Fig 3, Table 1). We also confirmed that 25-10-3 carried the exogenous chromosome that belongs to homologous group 6 of wheat.

**Table 1:** *P. huashanica* Ns genome SCAR markers and the wheat EST-SSR and EST-STS markers used in this study.

Marker	Accession No.	Primer sequence (5'-3')	Annealing temperature (°C)	Location
RHS141	HR614226	F: CTCGGCACCATAAACTAT	60	1-7Ns
		R: CTCGGCACTAGAGGAAAC		
RHS23	HR614210	F:ACGCAGGCACGTTCTGATGACTACT	69	1-7Ns
		R:ACGCAGGCACCAAATAACAATTATT		
BE403550	BE403550	F-ATGTTCTGTGTGGTGTCGGA	60	6AS 6BS 6DS
		R-ACATTGGGGAACAACTTGGA		
BE490147	BE490147	F-ACAGGAGGGTCACCAAGATG	60	6AL
		R-TGGAGCTGTCGTAGTGGTTG		
BE500768	BE500768	F-TTGTGGTTGATGGCAACACT	60	6AS 6BS 6DS
		R-GCTTTGTTCCCACGAGGATA		
BF201597	BF201597	F-AAAGGGAGGCTCTTCTGAGG	60	6AS 6BS 6DS
		R-GCTGAGCAGTATAGGCCAGG		
CD452568	CD452568	F: TTTGCATTTTCGTCTGCAAG	60	6AL 6BL 6DL
		R: TCGACACGAGCAAGATTCAC		
SWES2	SWES2	F: TGGACCCCGAGCATAACA	60	6A 6B 6DS
		R: CCCCAACACCGCAATCTA		

The marker Swes2 was mapped previously by Chen et al. (2005) and verified in the present study. The EST accession numbers were obtained from the following database: http://wheat.pw.usda.gov/SNP/new/pcr_primers.shtml

### Spike primordium differentiation

**Fig 4:**
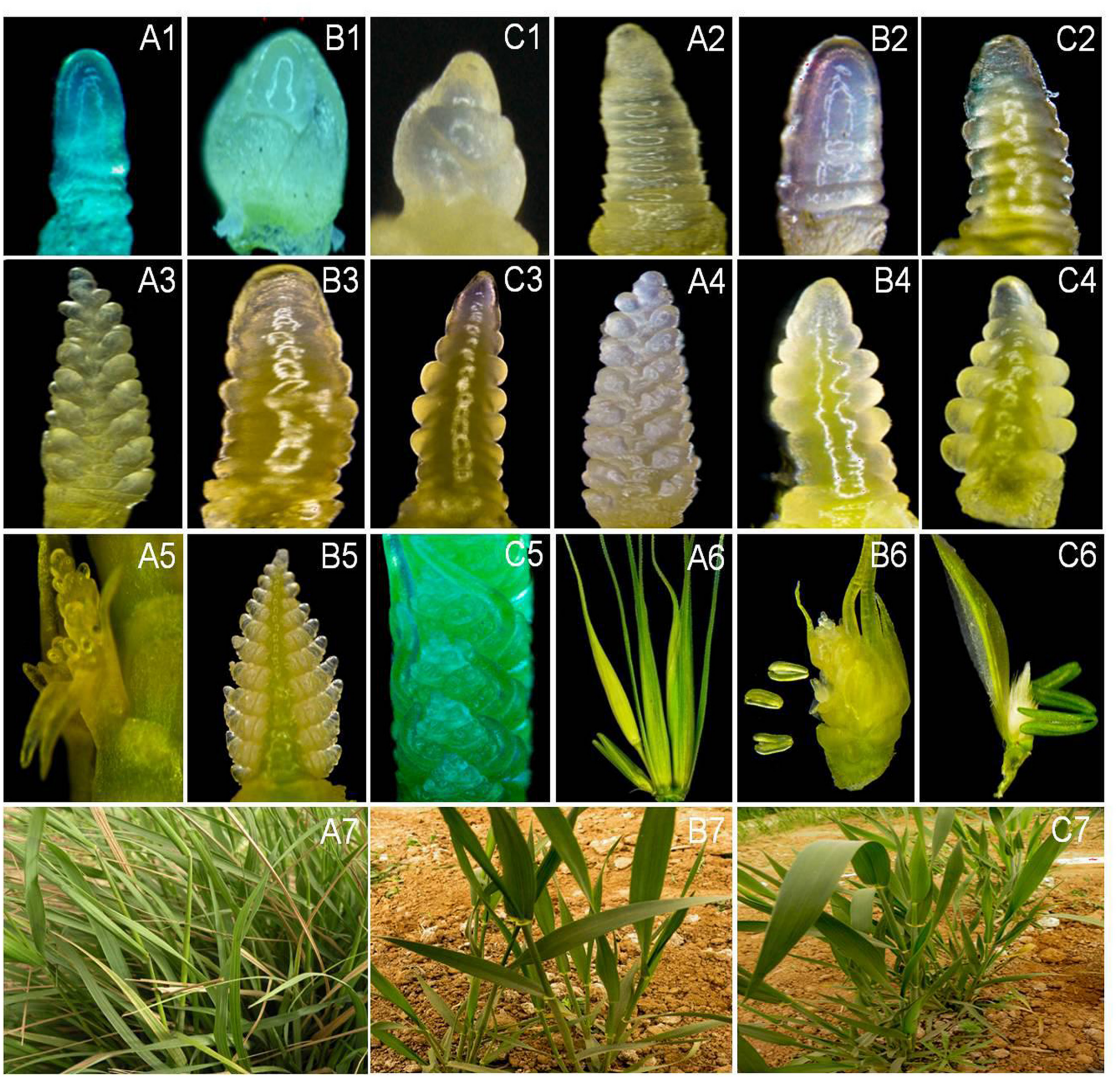
Anatomical observations of spike primordium differentiation obtained by binocular dissecting microscopy for addition line 25-10-3 and its parents, i.e., common wheat cv. 7182 and *P*. *huashanica* on different dates. A1–C1: November 6, 2011; A2–C2: December 6, 2011; A3–C3: January 6, 2012; A4–C4: February 6, 2012; A5–C5: March 6, 2012; A6–C6: April 6, 2012; A7–C7: April 6, 2012. A: *P*. *huashanica*, B: common wheat cv. 7182, C: addition line 25-10-3.

**Fig 5:**
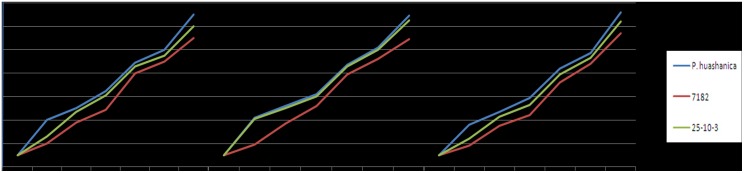
Spike primordium differentiation process. Horizontal axis: sampling date (October 2010 to July 2013), vertical axis: spike primordium development stage. 1: spike primordium; 2: single ridge; 3: early double ridge; 4: mid-double ridge; 5: later double ridge; 6: lemma primordium; 7: floret primordium differentiation; 8: pistil and stamen primordium differentiation; 9: green anther stage; 10: white anther stage; 11: flowering stage; 12: grain-filling stage; 13: maturity stage.

For three years (October 2010 to June 2013), we performed continuous microscopic observations of the spike primordia from sowing until the harvest, which showed that the spike primordia of these line exhibited clear differences throughout the entire growth period, particularly before the floret primordium stage where the maturation of 25-10-3 occurred about 10–14 days earlier than that of common wheat cv. 7182 (Figs 4 and 5). During early November in each year (Fig 4A1–4C1), 7182 and 25-10-3 were generally at the single ridge stage, which lasted 10–15 days, whereas *P*. *huashanica* was already in the later single ridge stage, and it grew faster than both common wheat cv. 7182 and 25-10-3. Therefore, there was no significant difference between common wheat cv. 7182 and 25-10-3 during this period. However, clear differences became apparent in early December (Fig 4A2–4C2), where common wheat cv. 7182was at the later single ridge stage whereas 25-10-3 developed faster than common wheat cv. 7182, and it was at the middle or late double ridge stage. During this period, we clearly observed that the spike primordium of the tiller entered the early single ridge stage. At the beginning of this period, the rate of spike primordium development decreased and it was the longest spike primordium differentiation process. *P*. *huashanica* developed to the late double ridge stage. During January (Fig 4A3–4C3), common wheat cv. 7182 was at the middle or later double ridge stage whereas 25-10-3 and *P*. *huashanica* entered the lemma primordium stage, although the terminal spikelets of 25-10-3 were still at the double ridge stage, after which all of the materials entered the overwintering period. The spikelet number and holding duration had a close relationship, where a longer time correlated with more spikelets, which is a key factor that contributes to high yields. During February (Fig 4A4–4C4), common wheat cv. 7182 differentiated into the lemma primordium stage but its terminal spikelets were still at the double ridge stage, while all of the terminal spikelets had differentiated into the lemma primordium stage in 25-10-3, whereas the apical meristem shoots had differentiated into spikelet meristem in *P*. *huashanica*. During March (Fig 4A5–4C5), common wheat cv. 7182 had entered the pistil and stamen primordium differentiation stage, whereas 25-10-3 and *P*. *huashanica* entered the floret primordium differentiation stage, and three stamens could be observed. During April (Fig 4A6–4C6; Fig 4A7–4C7), common wheat cv. 7182 moved onto the white anther stage and its flag was extended incompletely, whereas 25-10-3 and *P*. *huashanica* had moved onto the green anther stage and the flag leaf had extended to 5–10 cm. Thus, the differences between spike primordium development in these three test materials were mainly related to the single ridge to glume differentiation stages, which led to subsequent variation. The three materials of growth period and harvesting date from 2010 to 2013 was statistics respectively (Table 2).

**Table 2:** Growth period of wheat-*P*. *huashanica* addition line 25-10-3 and its parents, common wheat cv. 7182 and *P*. *huashanica*.

Material	2010.10–2011.6		2011.10–2012.6		2012.10–2013.6	
	Growth period (day)	Harvesting date	Growth period (day)	Harvesting date	Growth period (day)	Harvesting date
*P*. *huashanica*	212	May.14	215	May.17	208	May.11
7182	228	June .4	231	May .29	227	June.1
25-10-3	215	May.24	221	May.17	213	May.16

### Agronomic traits of disomic addition line 25-10-3 and its parents

The agronomic traits were tested for three consecutive years, which showed that common wheat cv. 7182 had an average plant height of 79.4 cm and a spike length of 8.2 cm, whereas addition line 25-10-3 was 66.24 cm high with a spike length of 7.8 cm, and *P*. *huashanica* was 77.6 cm high with a spike length of 7.6 cm. The spike length did not differ significantly as the plant height decreased. Compared with common wheat cv. 7182, 25-10-3 produced more grains per spike, more spikelets per spike, more kernels per spike, and it had a higher thousand-grain weight, thereby indicating that 25-10-3 probably carries beneficial genes that affect agricultural production (Table 3).

**Table 3:** Photosynthetic indices of wheat- *P*. *huashanica* addition line 25-10-3 and its parents, common wheat cv. 7182 and *P*. *huashanica*.

Photosynthesis index	Photosynthesis rate (*Pn*)	Stomatal conductance (*Gs*)	Transpiration rate (Tr)	Intercellular CO_2_ concentration (*Ci*)
	(μmol/(m^2^·s))	(μmol/(m^2^·s))	(μmol/(m^2^·s))	(μmol/(m^2^·s))
*P*. *huashanica*	11.19±0.53 Aa	0.09±0.07 Aa	238.94±4.14 Aa	2.92±0.71 Aa
7182	17.69±2.13 Bb	0.21±0.04 Aa	246.37±7.37 Aa	4.26±0.51 Aa
25-10-3	22.13±0.02 Bc	0.31±0.11 Ab	274.05±3.76 Aa	3.17±0.96 Aa

Significant differences in means are indicated at the *P* < 0.01 (capital letters) and *P* < 0.05 (lowercase letters) levels, according to Student’s *t*-tests.

### Photosynthetic indices

The photosynthesis-related indices, i.e., the photosynthesis rate, stomatal conductance, transpiration rate, and intercellular CO_2_ concentration, were determined during the grain-filling period, which showed that 25-10-3 had a significantly higher photosynthesis rate (reach 22.13±0.02μmol/(m^2^·s) compare with common wheat cv. 7182(17.69±2.13μmol/(m^2^·s); also, 25-10-3 (0.31±0.11 (μmol/(m^2^·s))) had a significantly higher stomatal conductance than common wheat cv. 7182 (0.21±0.11 (μmol/(m^2^·s)) (Table 4).

**Table 4:** Agronomic traits of wheat-*P*. *huashanica* addition line 25-10-3 and its parents, common wheat cv. 7182 and *P*. *huashanica*.

Characteristic	*P*. *huashanica*	7182	25-10-3
Plant height (cm)	77.60±2.70 Aa	79.40±4.16 Aa	66.24±1.79 Bb
Tillering	clump	8.20±1.92 Aa	8.40±1.14 Aa
Spike length (cm)	7.60±1.14 Aa	8.21±1.30 Aa	7.83±0.76 Aa
Spikelets per spike	15.40±1.82 Aa	17.60±2.41 ABab	20.00±0.71 Bb
Florets per spikelet	3.20±0.45 Aa	3.60±0.89 ABab	4.20±0.45 Bb
Kernels per spike	39.20±5.72 Aa	46.80±5.12 ABab	53.60±4.34 Bb
Thousand-grain weight (g)	3.56±0.23 Aa	37.50±1.40 Bb	41.52±2.19 Cc
Awn length (cm)	0.57±0.63 Aa	5.82±0.43 Bb	5.88±0.35 Bb

Significant differences in means are indicated at the *P* < 0.01 (capital letters) and *P* < 0.05 (lowercase letters) levels, according to Student’s *t*-tests.

## Discussion

Distant hybridization refers to the hybridization between different species, genus, and even further related species to combine their characteristics, properties and breakthrough species boundaries, so as to enlarge the genetic variation and create new variation types or new species. Distant hybridization of wheat is of great importance for the improvement of agronomic traits in common wheat via the transfer of alien chromosomes or fragments, such as yield and stress resistance in current wheat cultivars [17–20]. The production of wheat addition lines is the first step during the introgression of alien chromosomes into the host, but this approach cannot obtain translocation lines directly; thus, analyses must be performed to determine whether different alien chromosomes function in the complete wheat genetic background [21–23].

### Alien chromosomes affect the spike primordium development

We examined 25-10-3, a specific 6 Ns wheat-*P*. *huashanica* disomic addition line, to determine the effects of the added 6Ns chromosomes on the spike primordium initiation characteristics in field conditions and in laboratory experiment for three consecutive years. We found that its maturation period was about 10–14 days earlier than common wheat cv. 7182, especially from the single ridge stage and double ridge stage until the glume stage. Previous studies have demonstrated that this period is vital for the formation of large spikes and more florets in wheat, thereby obtaining a relatively favorable high yield in the following year [24,25]. In this study, the spike lengths and florets did not differ significantly, but the thousand-grain weight compensated for this deficiency. In a previous study, Farkas et al(2013). tested the addition of different H chromosomes (barley) and different wheat-barley disomic addition lines, which showed that the heading date and flowering time varied in different environments, but the 7H addition line was always the earliest in both a controlled environment test and field conditions [26]. Efremova et al. (2006)demonstrated that the ear emergence time and response to vernalization varied to different degrees in 12 5R(5A) alien substitution lines [27]. Thus, the 5R chromosome from rye, 7H from barley, and 6Ns from *P*. *huashanica* may have similar effects during the flowering period. The effects of early maturation may be important for the cropping practices in the Yellow-Huai River valley winter wheat zone and the low valley of the Yangtze River winter wheat zone in China. The duration of growth and maturity must be considered during wheat breeding, especially in areas of China where double cropping systems are applied each year, such as the Yellow-Huai River valley winter wheat zone (at 114–121° E, 32–40° N, where the annual average temperature was 14–15°C; the accumulated temperature ≥0°C and ≥10°C were about 4,100–5,400°C and 3,700–4,700°C, respectively; and the annual precipitation was 500–900 mm. Yangling Weather Burea supplied, Shaanxi, China) and the low valley of the Yangtze River winter wheat zone (at 110–120° E, 28–33°N, where the annual average temperature was 14–18°C, the accumulated temperature ≥10°C was about 4,500–5,000°C, and the annual precipitation was 1000–1400 mm, which occurred mainly in the spring and summer. Yangling Weather Burea supplied, Shaanxi, China). In particular, the first season crop is winter wheat in this system and the harvest season often experiences droughts with high temperature, or rain and preharvest sprouting, during this period [8]. Thus, harvesting the crop safely and avoiding these problems in the warmer summer can be difficult issues, which must be considered when planting, as well as by breeders. The temperature (accumulated temperature) is related to spike primordium development. The accumulated temperatures of the flowering days were recorded between October 2010 and June 2013. The growth of 7182 required an average accumulated temperature of 1295.6°C, where the effective accumulated temperature occurred with about 190–199 days until flowering. By contrast, the average accumulated temperature required for the growth of addition line 25-10-3 was about 1025.7°C with about 180–188 days until flowering. There was a difference of at least 10–14 days in the flowering times of 25-10-3 and common wheat cv. 7182, which was a highly significant difference. Early maturity could influence the planting patterns employed for double cropping each year, although it would have little impact on the yield and quality, but it could lead to more suitable conditions for the growth of the next crop. Thus, it might facilitate efficient crop management to obtain high yields throughout the year.

### Effects of the addition of 6Ns on common wheat cv. 7182

A high rate of photosynthesis is one of the basic characteristics of high yield materials. In the present study, photosynthesis-related indexes, i.e., the photosynthesis rate, stomatal conductance, transpiration rate, and intercellular CO_2_ concentration, were measured in the general experiment and they were correlated [28]. The photosynthesis rate is a characteristic of high-yield wheat and it is controlled mainly by the genotype, but also by environmental effects [29]. There have been many successful examples of the use of closely or distantly related species in distant hybrid breeding. For example, Chinese spring-*Lophopyrum elongatum* disomic substitutions, i.e., 2E^e^(2A) and 4E^e^(4A), possess a high photosynthesis rate from the heading to grain-filling stages [30]. Chen et al.(2013) also found that 6E^e^ had a positive effect on the photosynthesis rate [31]. Although *P*. *huashanica* had relatively low levels for all of the indices described above, the 6Ns addition line 25-10-3 had higher photosynthesis and stomatal conductance rates. This suggests that the 6Ns addition from *P*. *huashanica* affected the balance of photosynthesis in common wheat cv. 7182, or it contributed to a higher photosynthetic rate [32].

In conclusion, the addition of *P*. *huashanica* 6Ns chromosomes significantly affected the floral initiation and flowering times of wheat in field conditions. However, compared with other wild relatives of wheat, *P*. *huashanica* is still underutilized in terms of stress resistance. *P*. *huashanica* grows in an extreme environment, which is infertile, cold, and dry, but this species thrives in these conditions. Thus, we suggest that *P*. *huashanica* is an invaluable source of genetic material that carries various resistance-related genes, which are generally considered to be the main requirement for wheat breeding in practice. We consider that appropriate studies and the identification of new race-specific resistance genes will increase the utilization of *P*. *huashanica* as a promising genetic resource for wheat improvement. We plan to use these additions to develop wheat-*P*. *huashanica* translocation lines, thereby studying the effects of various genes located on smaller chromosome segments on the flowering time and other traits. The 6Ns chromosomes from *P*. *huashanica* were present in the addition line, but its agronomic characteristics differed from those demonstrated in previous studies, i.e., early maturation and a higher photosynthetic rate. Thus, it possible that the addition of the 6NS chromosomes was accompanied by another small segment or some genes may have introgressed and recombined with the wheat chromosome. Alternatively, the Ns chromosomes might have undergone degradation or recombination, but it was not possible to detect these changes using molecular markers and GISH. The agronomic traits of the addition line were clearly improved, i.e., its height, length, and thousand-grain weight. Thus, these distinct and stable economical characteristics should be considered during the selection of hybridization progeny.
